# Do women consult more than men? A review of gender and consultation for back pain and headache

**DOI:** 10.1258/jhsrp.2010.009131

**Published:** 2011-04

**Authors:** Kate Hunt, Joy Adamson, Catherine Hewitt, Irwin Nazareth

**Affiliations:** MRC Social and Public Health Sciences Unit, University of Glasgow, Glasgow; 1 Department of Health Sciences, University of York, York; 2 University College London, London, UK

## Abstract

**Objectives:**

Because women consult their general practitioners more frequently on average than men, it is commonly assumed that they consult more for *all* symptoms and conditions. This assumption is reinforced by qualitative studies reporting a widespread reluctance to consult by men. However, few studies directly compare consultation in men and women experiencing similar symptoms or conditions.

**Methods:**

A systematic review of the evidence on gender and consultation for two common symptoms, back pain and headache. Extensive electronic searches identified 15 papers reporting the relationship between gender and help-seeking for back pain and 11 papers for headache. Two independent reviewers assessed articles for inclusion and extracted data from eligible studies.

**Results:**

Few studies compared consultation patterns for these symptoms among men and women known to have experienced the symptom. The quality of the studies was variable. Overall, evidence for greater consultation by women with back pain was weak and inconsistent. Among those with back pain, the odds ratios for women seeking help, compared with men, ranged from 0.6 (95% confidence intervals 0.3, 1.2, adjusted only for age) to 2.17 (95% confidence intervals 1.35, 3.57, unadjusted), although none of the reported odds ratio, below 1.00 was statistically significant. The evidence for women being more likely to consult for headache was a little stronger. Five studies showed a statistically elevated odds ratio, and none suggested that men with headache symptoms were more likely to consult than women with headache symptoms. Limitations to the studies are discussed.

**Conclusion:**

Given the strength of assumptions that women consult more readily for common symptoms, the evidence for greater consultation amongst women for two common symptoms, headache and back pain, was surprisingly weak and inconsistent, especially with respect to back pain.

## Introduction

Several data sources suggest that women make higher use on average of primary care than men. For example, in the United Kingdom (UK) where most health care is free at the point of delivery under the British National Health Service, women consult their general practitioner (GP) more often than men, particularly in the peak reproductive years.^1^ However, most studies which have reported a female excess of consulting have used general practice or general population data which have not taken account of underlying symptomatology: as we have noted earlier, few studies^2^ examine consulting rates among men and women known to have comparable morbidity.^3^

The assumption that women are more ready to consult than men has been widespread for many years,^4–7^ and has lead to increasing interest in help-seeking behaviours among men.^8–11^ Courtenay has drawn a direct link between denial of weakness and rejecting help as key practices of masculinity and help seeking behaviour. He argues that:

By dismissing their health care needs, men are constructing gender. When a man brags, ‘I haven't been to a doctor in years’, he is simultaneously describing a health practice and situating himself in a masculine arena (p1389).^12^

Thus, it is often taken as a given that men ‘under-use’^13^ health care services: statements such as that ‘men are less likely than women to actively seek medical care when they are ill, choosing instead to “tough it out”’ (p47),^14^ are common within the literature. Such statements have contributed to a ‘strong public narrative … about health being “women's business” … leading to expectations that rather than seek help, men will be strong, stoical and often silent in matters relating to health and well-being’ (p112).^15^

Men's apparent reluctance to consult has been cited as ‘an important obstacle to improving men's health’ (p1058),^9,16^ fuelling concerns that fewer visits to the doctor and delays in seeking help may decrease men's chances for early detection, treatment, and prevention of disease. This message is frequently reinforced in sources of medical information. For example, a recent news item in the *British Medical Journal* elided assertions about gender differences in the use of health care with gender differences in mortality:

Health professionals … should move primary care into the workplace to make it easier for men to access health services, a debate held by the Men's Health Forum heard this week. Men currently use healthcare services far less effectively than women, and a recent Cancer Research UK survey found that men are almost 40% more likely than women to die from cancer^17^

It has been suggested that there is a ‘large body of empirical research support[ing] the popular belief that men are reluctant to seek help from health professionals’ (p5).^18^ There is indeed plenty of evidence from qualitative research suggesting that men do commonly vocalize a reluctance to seek help with symptoms of ill-health. However, most of this research is based on male-only samples and so cannot compare men's and women's discussions of their help-seeking behaviours), and some more recent studies^8,19^ have begun to highlight important exceptions to this common view.^10^

One of the relatively few general population studies which have compared consultation patterns in men and women with similar conditions concluded that their findings:

argue against the most widely accepted explanations for gender differences in consulting, namely that, once illness is recognised, women are more likely to consult than men (p98).^3^

As men's ‘under-usage’ of the health care system is constructed as a social problem, there is a danger that a contrasting presumption that women ‘overuse’ health care, consulting sooner and more often, sometimes for trivial symptoms which are self-limiting or amenable to self-management, is reinforced.^11^

The widespread assumption that men consult more readily than women needs to empirically challenged and verified, refuted or refined if best use is to be made of valuable health care resources. With this in mind, we have undertaken a review of literature (using systematic methods) on gender and consultation for two symptoms (back pain and headache). These symptoms were selected because: they are common within the population and account for a significant work load within health services; they are physically and socially disruptive for the individual, but rarely are an indication of serious life-threatening conditions. By reviewing the literature on consultation for headache and back pain, among people who have reported that they have these symptoms, we are able to examine studies that compared the proportions of men and women who were users or non-users of health care for headache and back pain.

## Methods

We used medical subject headings and text words to search several databases: Medline (Ovid; 1950 to October 2008), Embase (Ovid; 1980 to October 2008), PsychINFO (Ovid; 1806 to October 2008), CINAHL (Ovid; 1980 to October 2008), Social Science Citation Index (1956 to October 2008), Science Citation Index (1900 to October 2008), CDSR & DARE (Cochrane library – Issue 4 October, 2008), ASSIA (1987 to October 2008) and Sociological abstracts (1952 to October 2008). The literature search strategy was supported by Centre for Reviews and Dissemination, University of York. Health care consultation is found within the MESH term ‘Patient acceptance of health care’ which was used in the searches in combination with other keywords. The selection of keywords was informed by the search strategy used in a recent systematic review of access to health care.^20^ The search terms for consultation were then combined with the suite of terms identified for headache and back pain respectively. Appendix 1 provides detailed description of the search strategy used for Medline as an example. The search strategies were amended appropriately for each of the different search engines utilized. Where electronic copies of articles were available, use was also made of ‘cited by’ and ‘related article’ functions of the journals concerned. We also inspected reference lists of all included studies and of other relevant studies. In addition, we corresponded with key authors to attempt to identify any additional references.

### Selection of articles for inclusion

We considered only peer reviewed publications/abstracts that focused on consultation (definitions of consultation are discussed below) in response to symptoms of back pain and headache which included data on both users and non-users of health care. We restricted our review to studies which: included gender as an explanatory variable; were on adults; were conducted in developed countries; were based on observational epidemiological methods; and were published in English. Studies which did not specify the nature of the symptoms experienced and those which focused on referral patterns, repeated or frequent consultations, or which exclusively examined consultation with services outside of primary care were excluded.

Two reviewers (JA and either IN or CH) independently assessed electronic outputs (titles and abstracts) and the full-text articles of potentially relevant studies. Any disagreements were resolved by discussion between JA and IN/CH.

### Definitions of ‘consultation’

Any review of consultation behaviour in population-based studies is complicated by the lack of a common definition of help-seeking that is consistently operationalized within the literature. In this review, we have used the authors' own definitions of help-seeking. Our main interest was to examine help-seeking within primary care; however, initial screening of the literature demonstrated that very few studies had reported only on primary care consultation. Therefore, we broadened our definition of help-seeking to reflect how this had been used in the published studies. To maximize comparability between studies, we recorded help seeking as 1) overall consultation (if available), 2) consultation with a general practitioner only (if available). The time periods over which consultation was reported varied. To aid interpretation of patterns, we grouped studies which considered whether a participant had a) ever consulted for their symptom; b) consulted within the previous 12 months; c) consulted over some other time period.

### Data extraction and analysis

Two reviewers (JA and IN/KH/CH) extracted data on the study characteristics, definition of help-seeking, definition of back pain/headache and detailed information relating to the association between gender and help-seeking, in particular the odds ratio (OR) and 95% confidence interval (CI) for the relationship between gender and help-seeking (presented as OR for women compared to men). Where available we report the unadjusted and adjusted OR from the primary studies. In cases where only the adjusted OR was given in the paper, and where data were available, we calculated the unadjusted OR in order to be able to compare the two figures. For papers in which no ORs were presented for the impact of gender on help-seeking, we calculated the unadjusted ORs and 95% CIs where data were available. Calculations were performed using STATA 9. Given the heterogeneity of the primary studies, a meta-analysis was not appropriate in this instance.

In addition, we produced scatter plots of the associations between gender and help-seeking; if a publication included more than one OR in relation to gender and help-seeking (e.g. for different periods of consultation or before and after adjustment for other factors) (see tables), the more conservative estimate cited in the tables was plotted. Studies which had simply stated there was no association between gender and help-seeking (and did not include data which allowed calculation of an OR) were represented in the scatter plot as having an odds ratio of one. To aid interpretation of the results, studies are grouped by definition of consultation (ever consulted, consulted within previous 12 months, consulted over another time period) and within these groups by year of publication.

## Results

**Figure 1:**
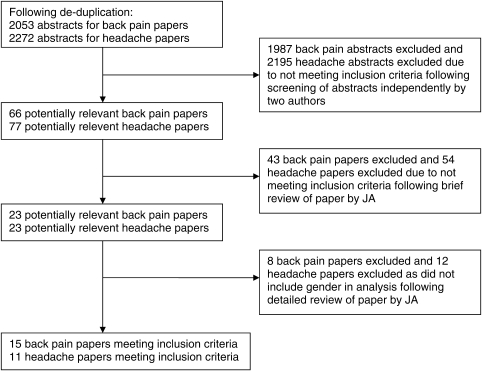
Progress of search for relevant papers

Figure 1 shows the number of back pain and headache articles that were initially identified (*n* = 2053 back pain; *n* = 2272 headache) and then screened out independently by two assessors.

### Gender and consultation for back pain

We identified just 15 eligible publications reporting the relationship between gender and consultation for back pain, all based on cross-sectional studies;^21–35^ the majority used general population samples, although four used workplace populations.^30,31,33,34^ The studies were conducted in a range of countries with differing health care systems, most (*n* = 9) in northern Europe and the USA (*n* = 4) and one each from Greece and Australia. The number of participants ranged from 54^22^ to 8316,^27^ and the percentage of men in the sample (where given or possible to calculate) varied from 12% (for a sample of care-workers)^31^ to 94% (for a sample of industrial workers).^30^ Definitions of symptom experience also varied considerably from reports of having ‘had back pain at least once’,^21^ to a ‘history of back pain,^26^ to use of the Nordic questionnaire.^36^

Definitions of consultation were also very inconsistent and varied both by type of service provider and time period considered. Most commonly studies described consultation with any health care provider. This varied across studies, but generally included primary and secondary care (including specialist services) and in some cases complementary and alternative resources (particularly chiropractors). Few papers referred specifically to primary care (*n* = 3). Most papers imposed a time-frame within their definition of help-seeking (most commonly the previous 12 months), but these ranged from ever having consulted for the back pain to consultation within the last month.

**Figure 2:**
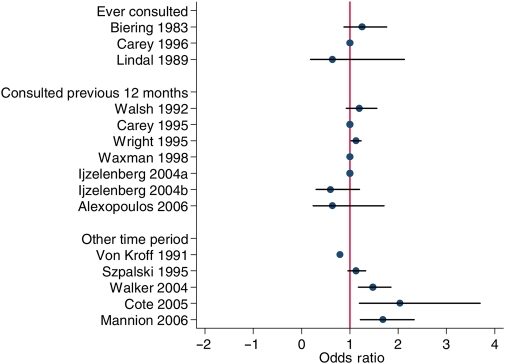
Scatterplot of association between gender and consultation for back pain by period of consultation (ever consulted, consulted in previous 12 months, other time period)

The ORs for women with back pain seeking help, compared to men with back pain, are shown in Table 1. The observed association between gender and help-seeking ranged from OR 0.6 (95% CI 0.3, 1.2, adjusted for age)^30^ to OR 2.17 (95% CI 1.35-3.57, unadjusted).^33^ However, this is across all definitions of help-seeking, and descriptions of symptoms. None of the studies which examined *ever* consultation for back pain symptoms showed any relationship with gender, nor did any of the seven studies which considered consultation *within the previous 12 months* (Figure 2). Among those which considered consultation over other time periods, three which considered shorter time periods – previous six months,^32^ within 4–16 weeks of reporting problem at work,^33^ last month^35^ – suggested that women consulted more than men. Hence, overall, evidence for greater consultation for back pain by women in comparison with men was weak (although no studies suggested that men were more likely to consult than women).

**Table 1:** Association between gender and help-seeking for back pain

Authors	Year	Country	*n*	% male in sample	Definition of back pain	Definition of help-seeking	OR (95% CI)
Biering–Sorensen^21^	1983	Denmark	575	48.9	Had low back pain at least once	Consulted a general practitioner at some time for low back pain	1.34 (0.96–1.88)*
						Consulted a health care provider at some time for low back pain (includes general practitioner, specialist or chiropractor)	1.25 (0.88–1.76)*
Lindal and Uden ^22^	1989	Sweden	54	42.6	Currently had back pain	Consulted a physician for current back pain problems	*0.64 (0.19–2.13)*
Von Korff et al**^23^	1991	US	411	Dna	Back pain within previous six months	Consulted a health care provider for back pain in the previous six months (includes doctor, physical therapist, chiropractor, or other health care professional)	0.8 NS** (adjusted for age, back pain onset >= five years, persistent pain, pain severity, self-rated health, psychological distress)
Walsh *et al.*^24^	1992	UK	963	45.9	Reported back pain in previous 12 months	Consulted general practitioner in the previous 12 months for low back pain	1.20 (0.93–1.56)* 1.3 (1.0–1.8) (adjusted for age, social class, disability score and area of residence)
Carey *et al.*^25^	1995	US	269	34	Chronic low back pain (functionally limiting back pain for >three months or >25 spells of back pain in previous year)	Consulted a health care provider in the previous 12 months for back pain (includes primary care doctor, chiropractor, physical therapist, orthopedic surgeon)	NS*
Szpalski *et al.*^26^	1995	Belgium	2660	43	History of low back pain	Visited physician or other health professional for the current or last episode of low back pain	1.13 (0.96–1.33)* NS (adjusted for daily low back pain, language, area of residence, social class, age, lifelong low back pain, good health)
Wright *et al.*^27^	1995	UK	8316	39.7	In the last 12 months have you suffered from sciatica, lumbago or recurring back pain	Visited a doctor in the last 12 months in response to sciatica, lumbago or recurring back pain	1.13 (1.03–1.23)*
Carey *et al.*^25^	1996	US	485	46	Acute severe low back pain (functionally limiting back pain lasting less than three months)	Ever consulted a health care provider for back pain (includes physician, chiropractor, physical therapist, nurse, massage therapist)	NS*
Waxman *et al.*^29^	1998	UK	540	Dna	Ever had back pain lasting more than a day in the previous 12 months	Consulted a health care provider in the previous 12 months for back pain (includes general practitioner, hospital doctor, workplace doctor/nurse, pain clinic or accident and emergency)	NS (adjusted for employment, diagnosed low back pain, pain started <one year ago, chronicity, pain, external locus of control)
Ijzelenberg and Burdof^30^	2004	Netherlands	305	12	Nordic questionnaire	Consulted general practitioner in the previous 12 months for low back pain	0.6 (0.3–1.2) (adjusted for age)
Ijzelenberg and Burdof^31^	2004	Netherlands	252	94.2	Nordic questionnaire	Consulted health care provider in the previous 12 months for low back pain (includes general practitioner, specialist or physical therapist)	NS*
Walker *et al.*^32^	2004	Australia	1228	45.4	Low back pain in previous six months	Consulted a health care provider in the previous six months for low back pain (includes general practitioner, chiropractor, massage therapist, physiotherapist)	1.48 (1.18–1.85)* 1.7 (1.3-2.2) (adjusted for pain/disability, fear, marital status, cause of low back pain)
Cote *et al.*^33^	2005	US	1104	49.2	Workers compensation claim form for work-related back pain	Consulted health care provider within 4-16 weeks of reporting the back pain problem at work (includes medical physican, physical therapist, chiropractor, osteopath, surgeon, accident and emergency, nurse, massage therapist, acupucture)	2.17 (1.35–3.57)* 2.04 (1.2–3.7) (adjusted for age, severity of injury, history of back pain, employer, occupation, job satisfaction, time from onset, propensity weights)
Alexopoulos *et al.*^34^	2006	Greece	314	Dna	Nordic questionnaire	Consulted a health care provider in the previous 12 months for low back pain (includes general practitioner, specialist, physiotherapist, occupational physician)	0.64 (0.24–1.71) (adjusted for chronicity)
Mannion *et al.*^#^^35^	2006	Switzerland	2507	Dna	Current lower back pain	Consultation with health care provider in the last month for low back pain (includes specialist, general practitioner, physiotherapist, or other practitioner)	1.69 (1.22-2.33) (adjusted for age, pain frequency, fear avoidance beliefs)

*Unadjusted

OR, odds ratio; Dna, data not available

ORs presented in italics have been calculated from percentages presented in the paper

Note: Nordic questionnaire^36^ is a standardised instrument valid for collecting data on the nature, duration and frequency of back pain symptoms

### Gender and consultation for headache

The 11 publications from which it was possible to extract information on gender and consultation for headache showed a similar range of methodological variability as did the studies of consultation for back pain (see Table 2).^23,37–46^ All the eligible studies on headache also employed a cross-sectional design, and most (*n* = 7) were conducted in the USA, with the remaining four papers from north European countries with different health care systems. Sample sizes ranged from *n* = 82^46^ to *n* = 9380,^37^ and the percentage of men in the studies (where given or possible to calculate) from 13.6%^40^ to 46.6%.^37^ Again definitions of headache symptoms varied, as did the type of service provider consulted and the time period.

**Table 2:** Association between gender and help-seeking for headache

Authors	Year	Country	*n*	% male in sample	Definition of headache	Definition of help-seeking	OR (95% CI)
*Celentano *et al.*^37^	1990	US	9380	46.6	One or more headaches in previous 12 months	Ever consulted a physician for headache	2.32 (2.08–2.58)*
						Consulted physician in past 12 months for headache	2.72 (2.34–3.16)*
			6347	39.3	One or more headaches in the previous four weeks	Consulted physician in past 12 months for headache	2.15 (1.79–2.58) (adjusted for age, pain and duration of most recent headache attack)
Von Korff et al.**^23^	1991	US	263	dna	Headache within previous six months	Consulted a health care provider for headache in the previous six months (includes doctor, physical therapist, chiropractor, or other health care professional)	1.1 NS (adjusted for age, headache onset >= 5 years, persistent pain, pain severity, self-rated health, psychological distress)
Rasmussen *et al.*^38^	1992	Denmark	697	42.5	IHS criteria for migraine/tension type headache (TTH) at any time	Ever consulted a general practitioner for headache	2.83 (1.91–4.20)*
Rokicki and Holroyd^39^	1994	US	190	18.9	Headaches more than five times per year and characteristic symptoms of tension or migraine headaches	Consult professional help for headache	1.40 (0.66–2.93)*
Ziegler and Paolo^40^	1996	US	104	13.6	Cases – attending headache clinic	Cases – attending headache clinic	NS*
					Controls – suffered frequent moderate or severe headache	Controls – not consulted a doctor in previous two years for headache	
Lipton *et al.*^41^	1998	US	1720	19.9	One or more migraine headaches in previous year (IHS criteria)	Ever consulted a doctor for their headaches	1.61 (1.26–2.04)*
Lipton *et al.*^42^	2002	US	242	15.0	Migraine (IHS criteria) and reported at least six headaches in previous 12 months	Ever consulted a doctor for headache	2.88 (1.42–5.84)*
						Consulted a doctor for headache in previous 12 months	1.86 (0.91–3.82)*
Lipton *et al.*^43^	2003	UK	143	15.4	Migraine (IHS criteria) and reported at least six headaches in previous 12 months	Ever consulted a doctor for headache	2.07 (0.70–6.27)*
						Consulted a doctor for headache in previous 12 months	1.22 (0.50–2.98)*
Lucas *et al.*^44^	2004	France	312	27.6	Experienced migraine (IHS criteria) with at least one attack in the previous three months	Ever consulted a physician for headache	1.60 (0.91–2.82)*
Thomas *et al.*^45^	2004	UK	1871	39	Experienced headache in previous three months	Consulted health care professional in previous three months for headache (including hospital specialist, general practitioner, dentist, optician, pharmacist)	1.45 (1.1–1.9)*
Skomo *et al.*^46^	2006	US	82	Approx 30**	Currently experiencing migraines (IHS criteria)	Consulted physician for headache in previous 12 months	NS (adjusted for age, migraine disability, social support, locus of control, beliefs about medication)

*Unadjusted

OR, odds ratio; Dna, data not available

**actual data not available, but able to make approximation

ORs presented in italics have been calculated from percentages presented in the paper

Note: International Headache Society (IHS) system is a widely accepted diagnostic tool for differentiating headache type and is considered the gold standard^48^

**Figure 3:**
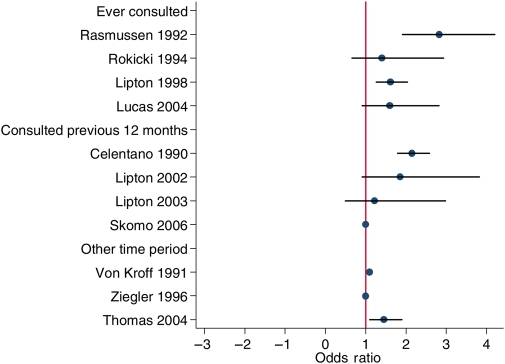
Scatterplot of association between gender and consultation for headache by period of consultation (ever consulted, consulted in previous 12 months, other time period)

Despite this plurality of design, there was more consistent evidence for greater consultation among women with headache symptoms than for men with similar symptoms (Figure 3). None of the 11 publications suggested that men were *more* likely to consult for headache (i.e. OR < 1.0), five studies suggested a statistically significant positive relationship, and the remaining six reported equivocal relationships between gender and consultation for headache. Two studies with positive relationships were found among the four which considered consultation ‘ever’, one among the four that considered consultation in the previous 12 months, and one in a study reporting experience of symptoms and consultation in the previous three months.

## Discussion

We undertook this review to assess whether there was evidence to support the widespread assumption that women are more ready to consult than men, not just in general but for specific conditions or symptoms. We chose to focus on two symptoms which are not usually life-threatening but which nonetheless cause distress and disruption of daily life for the sufferer, contribute to significant losses to the economy (for example, one study reported that 43% of migraine sufferers missed one or more days' work because of their headaches^38^), and are commonly presented to general practitioners,^47^ accounting for a substantial amount of NHS resources. Against the widespread assumptions that men are more reluctant to consult than women, our review did not reveal strong or consistent evidence of greater consultation among women for back pain than for men. The evidence that women consult more than men for headache was stronger, but by no means fully consistent. The ORs reported are generally lower than those reported in studies that were *not* able to take account of the presence of headache symptoms in non-consulters.^47^

We encountered a number of challenges in conducting the review including: the poor quality of some of the studies; the plurality of definitions of the underlying symptoms (back pain and headache); and the plurality of definitions of ‘consulting’, both with respect to type of health care provider and period of time considered. Although we restricted our review to studies which indicated that they included both men and women, and consulters and non-consulters, which theoretically allowed for the relationship between gender and consultation to be examined, often relevant details (such as the proportion of men and women in the sample, or the OR for consultation by gender) were difficult to extract. We have argued elsewhere^11^ that there is a need for more gender comparative research on help-seeking behaviours. This review illustrates the need for well-designed empirical studies which can address whether there are gender differences in use of health care.

It is important to note the methodological limitations of the studies included in this review. The studies we uncovered were largely cross-sectional in nature. Given the relatively homogenous methodological design across studies, the use of specified quality criteria (which lack consensus about which is most appropriate) would have rated all of the studies very similarly. We therefore, concluded that this would add little to the review. For example, while symptom measurement was often based on standardized tools (most commonly the Nordic questionnaire for back pain studies^36^ and International Headache Society (IHS)^48^) criteria for headache studies), the outcome measure was not. It is likely that all studies in the review were subject to recall bias; however, the different definitions of consultation (over different time periods) suggest that recall bias varied across studies. Most importantly for this review is the question of whether recall bias is likely to be systematically different for men and women. However, it is difficult to predict whether this would lead to systematic over- or under-estimation of health service usage.

Differences in help-seeking across gender may also have been masked in some studies through the combination of service types (e.g. primary, specialist care and complementary and alternative medicine) used in the definition of consulting as these might have different predictors. In addition, there are differences across studies in the extent of adjustment in the statistical analysis. We might have expected any association between gender and consultation to be attenuated when ‘need’ variables relating to the symptoms (for example, severity/duration/frequency/disability) and other sociodemographic characteristics were adjusted for. However, there were only four papers from the back pain review where both unadjusted and adjusted figures were available; in two cases the adjusted OR was attenuated and in two cases the adjusted figure was greater.

Given the heterogenity across the studies in this review we did not conduct a formal meta-analysis. We feel this would have projected a false sense of precision onto the data available (given the nature and quality of the primary studies). It may have also led to a biased estimate of the association between gender and consultation behaviour as it would not have allowed the inclusion of studies which merely stated there was ‘no association’ between gender and consultation but did not provide further data on this. There is also the potential problem of publication bias, whereby studies which do not demonstrate an association are less likely to be published. By including these studies in the review we may have minimized the potential for publication bias.

It is important to note too that the aim of many of the papers included in this review was to identify a range of factors that influenced consultation. Gender was never the sole factor examined, and was usually one of several characteristics studied (including other sociodemographic characteristics, symptom variables, psychological variables and social relationships). Many of the papers in this review were based on data that were largely opportunistic in nature. For example, many of the studies have addressed questions of care-seeking as secondary to prevalence studies of back pain/headache and were never designed to specifically address issues of consultation and hence relied on a single question about consultation within a large questionnaire.

The often unchallenged, but widespread, assumption that women will consult more readily for *all* symptoms or conditions and that men will be more reluctant or will delay consulting may result in health care providers assuming that women have a lower level of symptom severity before deciding to consult. This could affect (albeit unconsciously) these providers' assessments of the symptoms, their diagnosis and their strategy for helping a patient manage their symptoms. Sophisticated experimental studies which found that patient gender significantly influenced doctors' proposed management strategies support this view. When doctors were shown videotapes of identical presentations of symptoms which could be indicative of coronary heart disease, female ‘patients’ were asked fewer questions and were recommended for fewer diagnostic tests. Doctors were more likely to ‘tune into psychological cues and to search for psychological explanations for symptoms’ when the presenting patient was female.^49,50^

Attempts to summarize quantitative evidence on whether men and women make similar use of health care services (when comparing men and women with similar morbidity) should be set against what Connell describes as the ‘commonsense knowledge [that] men and women act differently’ (p4).^51^ This assumption of difference has become deeply entrenched in public and medical views of men's and women's use of primary care. If we are to best serve both women's and men's health service needs then such assumptions need to be carefully examined, contributing to an evidence-base that supports or refutes this commonsense knowledge. Only then will it be possible to identify the particular barriers that men and women face to the most effective and appropriate use of health services and other forms of help-seeking for different health problems. There is still a dangerous (often implicit) tendency to assume that, if men employ a public reluctance to seek help as one important way of demonstrating their masculinity, then this must necessarily suggest that women are *not* reluctant to seek help.

## Appendix 1: MEDLINE (Ovid) search strategy

**Table:**

#	Search History
**Backache**	
1	Patient acceptance of health care
2	Health services/ut
3	Attitude to health
4	Health behaviour
5	Health knowledge, attitudes, practice
6	Communication barriers
7	Professional–patient relations
8	Physician–patient relations
9	Health services needs and demand
10	Health services accessibility
11	or/1–10
12	Exp back pain
13	Backaches or back aches
14	Back pains
15	Vertebrogenic pain syndrome
16	or/12–15
17	11 and 16
18	Limit 17 to English language
**Headache / migraine**	
1	Patient acceptance of health care
2	Health services/ut
3	Attitude to health/
4	Health behaviour
5	Health knowledge, attitudes, practice
6	Communication barriers
7	Professional–patient relations
8	Physician–patient relations
9	Health services needs and demand
10	Health services accessibility
11	or/1–10
12	Headache
13	Headaches or head aches
14	Head pains or cranial pains
15	Hemicrania or cephalgia or cephalalgia or cephalodynia
16	Exp migraine disorders
17	Migraines or migrainous
18	or/12–17
19	11 and 18
20	Limit 19 to English language
